# Correction of the pelvic incidence using a bilateral extending pelvic osteotomy: a proof of concept study

**DOI:** 10.1007/s00402-022-04425-1

**Published:** 2022-04-07

**Authors:** A. E. A. Ochtman, R. L. A. W. Bleys, J. E. Cunningham, F. C. Öner, S. M. van Gaalen

**Affiliations:** 1 Department of Orthopedics, University Medical Center Utrecht, PO box 85500, 3508 GA Utrecht, The Netherlands; 2 Department of Anatomy, University Medical Center Utrecht, PO box 85500, 3508 GA Utrecht, The Netherlands; 3 Epworth Richmond, 89 Bridge Rd, Richmond VIC, 3121 Australia; 4 Acibadem International Medical Center, Arlandaweg 100, 1043 HP Amsterdam, The Netherlands

**Keywords:** Sagittal alignment of the spine, Bilateral extending pelvic osteotomy, Proof of concept, Cadaveric study

## Abstract

**Introduction:**

The aim of this proof of concept human cadaver study was to quantify the effect of a bilateral extending pelvic osteotomy (BEPO) on pelvic incidence (PI) as a potential alternative for a pedicle subtraction osteotomy (PSO) in patients with severe spinal sagittal malalignment.

**Materials and methods:**

10 fresh frozen human cadavers were treated with the BEPO technique. CT images were made before and after the osteotomy and pure sagittal images were created on which PI was measured.

**Results:**

The mean pre-osteotomy PI was 47.9° (range 36.4–63.9) and the mean post-osteotomy PI was 36.5° (range 22.1–54.4). The mean correction was − 10.4° with a range of − 8.4° to − 17.3° (*p* = 0.03), which resulted in a mean decrease of 23% in the PI (range 16–42).

**Conclusions:**

There was a feasible and effective correction of PI using the BEPO technique on the os ilium. This was a preliminary cadaveric study. No conclusions could be made on global sagittal alignment. We postulate that an extending osteotomy of the ilium could be a potential alternative for a PSO reducing the complexity of spine surgery in patients with severe spinal sagittal malalignment.

## Introduction

The pelvis is the pedestal for spinal alignment. The recognition of pelvic morphology as a regulator of global sagittal spinal alignment and determinant of spinal pathology is increasing [1–3]. As humans evolved towards bipedal posture, morphological changes of the pelvis were crucial. A fully erect bipedal posture with extended hips and knees without an adaptation of the pelvis would require an extreme lumbar lordosis (LL) to keep the trunk above the femoral heads [4]. Lordotic angulation between the ischium and ilium, quantified as the ischio-iliac angle (IIA), made humans able to stand upright with a relatively small LL. The IIA is strongly related to the PI [5], a morphological parameter defined as the angle between the line perpendicular to the sacral plate at its midpoint, and the line connecting this point to the axis of the femoral heads (Fig. 1) [2]. Both parameters are unique to each individual and stay constant after adolescence under normal circumstances. The sacral plate is the base of the spinal column and thus its position influences the degree of LL to maintain a balanced upright position. Low PI is associated with decreased LL and high PI with increased lordosis [6]. Schwab et al. formulated this as: LL = PI±9 [7].

**Fig. 1:**
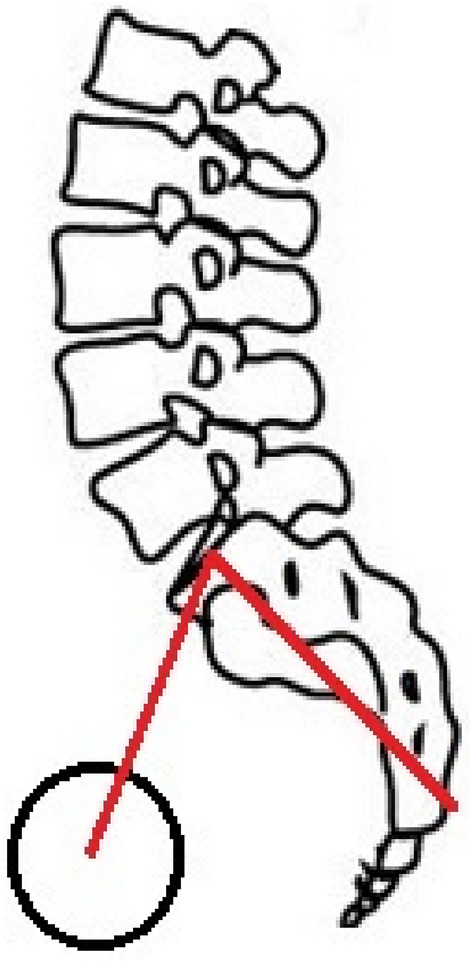
Pelvic incidence (PI): a morphological parameter defined as the angle between the line perpendicular to the sacral plate at it midpoint, and the line connecting this point to the axis of the femoral heads

Many disorders of the spinal column can lead to changes in this equilibrium. Because PI remains relatively constant during life, loss of LL may lead to a mismatch between PI and LL and increased pelvic tilt (PT). This loss of sagittal alignment is associated with reduced quality of life, especially increased PT [8–10]. In patients with a limited PI-LL mismatch, surgical correction can be achieved by restoring disc height with a variety of interbody surgical techniques. In case of severe loss of LL, restoration can only be achieved by a comprehensive correction osteotomy such as multiple Smith-Petersen, Ponte- or pedicle subtraction osteotomies (PSO), according to the SRS-Schwab Radiologic Classification [11]. These procedures, however, are associated with many complications, such as blood loss, deep wound infection and neurological deficits, especially in patients with previous lumbar spinal surgery [12].

An alternative place for an osteotomy could be between the sacral plate and the femoral heads, to decrease PI. Based on the correlation between PI and the IIA, we hypothesize that correction of these parameters may reduce the need for more drastic osteotomies in the lumbar spine. Similar pelvic osteotomies such as Salter [13] and Chiari [14] have been well described for the treatment of hip dysplasia. However, these are performed in young children and are mostly performed on one side. The effects of a bilateral osteotomy on restoration of spinal sagittal malalignment has received little attention thus far [15, 16]. The aim of this study is therefore to quantify the anatomical effects of bilateral anterior open-wedge correction osteotomies of the ilium (further referred to as bilateral extending pelvic osteotomy (BEPO)) on PI in human cadavers.

## Materials and methods

### Specimen preparation

We included 10 human cadavers (mean age 74.3 years, range 54–92) in this study. In accordance with Dutch legislation, the bodies were donated and destined for medical education and research to the Department of Anatomy of the University Medical Center Utrecht by last will. Body handling was done according to the guidelines of the Department of Anatomy. None of the deceased subjects had any history of pelvic or hip surgery. The freshly frozen (− 20°) cadavers were thawed 24 h before the pelvis and sacrum were harvested. The pelvic soft tissues were carefully removed to improve visibility of the bone, especially fractures or fissures, and prevent soft tissue obstructing the most ideal correction. No ligaments were removed. Computer tomography (CT) scans were obtained before and after the BEPO procedure (iCT, 120 kV, slices 0.9 mm, Philips, Eindhoven, The Netherlands).

### BEPO surgical technique

Each pelvis was positioned supine. With a thin oscillating saw blade, a straight cut was made starting from the area in between the anterio-superior and anterio-inferior iliac spine and targeting the greater sciatic notch. The same cut was performed on the contralateral side. In the first five specimens, the saw cut ended at the arcuate line of the ilium, approximately two thirds of the length between the iliac crest and the sciatic notch. For specimen 6 to 10, the surgical technique was improved by making the saw cut past the arcuate line (approximately 80% of the length between iliac crest and sciatic notch) to prevent a potential fracture during osteotomy distraction. The thin blade osteotome was gently placed in the osteotomy gap to measure the angle when the tip of the instrument reached as close to the hinge point as possible. The insertion depth corresponded with the previously sawn cut. A second blade osteotome was then slowly inserted to the same depth. A third blade was then inserted between the previously inserted blades. Then, a TomoFix Bone spreader with 8 mm blade (range 6–20 degrees) was inserted and a hexagonal screwdriver was used to open the spreader to the desired 15 degrees level. This technique is based on the medial open-wedge tibial osteotomy described for the TomoFix plate [11, 17]. This first osteotomy was kept open with bone spreader forceps. The same procedure was then performed on the contralateral side. Next, two TomoFix bone distractors (Johnson and Johnson Services Inc., New Brunswick NJ, USA) were carefully hammered into both osteotomies until the hinge was reached. The screws of the TomoFix were slowly turned with a screwdriver to gently spread the osteotomy to the desired opening angle of 15 degrees. To maintain the wedge, a PLIF cage size 15 (EIT Emerging Implant Technologies Inc., Tuttlingen, Germany) was inserted (Fig. 2).

**Fig. 2:**
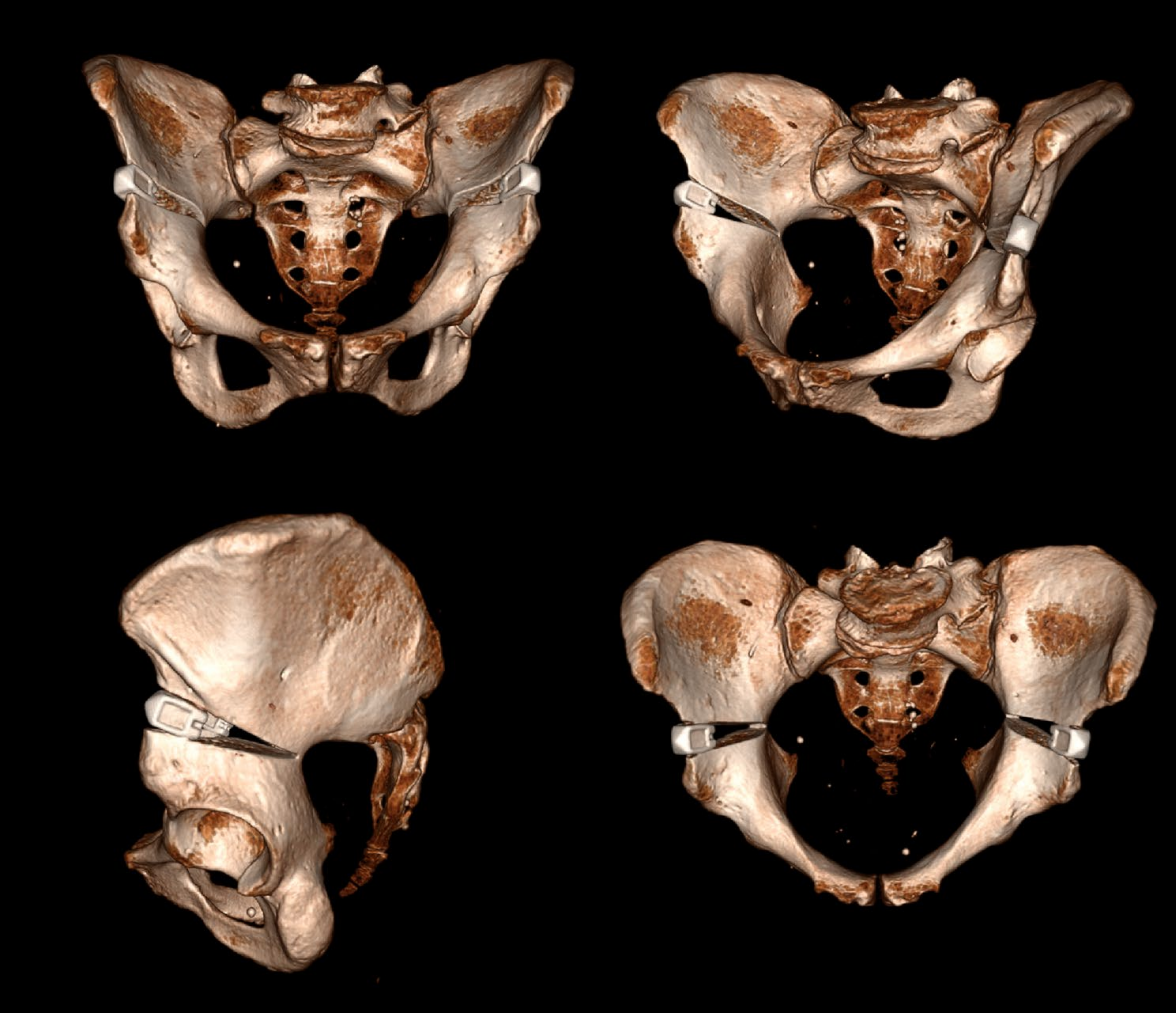
3D reconstruction of CT scan: AP, ¾, lateral and proximal view

### Data and statistical analysis

CT images were uploaded in the Picture Archiving and Communication System (PACS) of the University Medical Center Utrecht (ChipSoft Inc., Amsterdam, The Netherlands). With multiplanar reconstruction (MPR), pure sagittal reconstructions were created, with both acetabular bones projected exactly overlying each other. These reconstructed images were imported into the previous validated software Surgimap^®^ (Nemaris Inc., New York, NY, USA) [15]. To measure PI, the specialized sagittal alignment tool was used where acetabulum and the superior endplate S1 were identified after which PI was automatically generated by the software. Each image was measured 4 times (3 times by the first author on three consecutive days, one by one other observer). Measurements were used to calculate the mean absolute difference.

Statistical analyses were performed with Statistical Package for the Social Sciences software (SPSS 23.0, SPSS Inc., Chicago, IL, USA) using the Wilcoxon signed-rank test and Mann–Whitney *U* test. Inter-rater and intra-rater reliability was assessed using intra-class correlation (ICC) coefficients. Statistical significance was set at *p* < 0.05.

## Results

An overview of the demographics of the specimen and the results of the osteotomy on PI are presented in Table 1. We found BEPO to be considerably effective in changing PI. The mean pre-osteotomy PI was 47.9° (range 36.4–63.9) and mean post-osteotomy PI was 36.5° (range 22.1–54.4). The mean correction was − 10.4° with a range of − 8.4° to − 17.3° (*p* = 0.03), which resulted in a mean decrease of 23% (range 16–42). Both inter- and intra-reliability analyses revealed excellent agreement for PI measurements (ICC = 0.92, *p* = 0.004 resp. ICC = 0.97, *p* = 0.001).

**Table 1:** Overview of specimen and effect of BEPO on PI

Specimen	Sex	Age (y)	Preoperative PI	Postoperative PI	Correction (%)	
1	Female	85	37.5	28.6	− 8.9 (24)	
2	Female	68	59.4	49.8	− 9.6 (16)	
3	Female	89	36.4	27.6	− 8.8 (24)	
4	Male	54	50.5	35.4	− 15.1 (30)	
5	Male	58	43.9	35.5	− 8.4 (20)	
6	Female	70	39.4	22.1	− 17.3 (42)	
7	Male	75	63.1	47.5	− 15.6 (25)	
8	Male	92	44.1	34.1	− 10.0 (23)	
9	Female	83	63.9	54.4	− 9.9 (16)	
10	Female	69	41.3	30,1	− 11,2 (27)	
Mean		74.3	47.9	36.5	− 10.4 (23)	*p* = 0.03

A sub analysis was performed on specimen 1–5 (group A) and specimen 6–10 (group B), because of the slight adjustment in the surgical technique. The mean correction in group A was − 10.2° (*p* = 0.04) and in group B − 12.7° (*p* = 0.04). When comparing both groups, no statistically significant difference was found (*p* = 0.46).

### Technical complications

In specimen no. 1 and no. 3, a small unilateral hairline fracture occurred when the opening wedge was created with the bone distractor. However, these fractures did not cause any dislocation and the stability was not reduced as a result, thus the clinical relevance has yet to be established.

In specimen no. 4, a unilateral fracture occurred that did have impact on the stability and would have needed additional fixation (osteosynthesis) in clinical setting. After refinement of the surgical technique, no further fractures were observed when the saw cut was made beyond the arcuate line.

## Discussion

The increased recognition of the importance of sagittal spino-pelvic alignment has led to the consideration that the pelvis could be seen as pelvic vertebra, as suggested before by Dubousset [18]. Spinopelvic parameters, such as IIA, PI and LL are strongly correlated with each other [19]. These patterns are essential in understanding the differences in biomechanical loading of the spine and etiology of spinal pathology such as degenerative disc disease and spondylolisthesis and the influence on sagittal spinal malalignment and therefore its surgical treatment.

In the present study, we quantified the effect of a BEPO on PI in human cadavers. We found a statistically significant decrease of PI to prove the concept. The effect on the global sagittal alignment and the clinical relevance has yet to be established. We speculate that this may diminish the need for complex PSO surgeries with concurrent complications and increase the need for less complex procedures such as SPO or the use of hyperlordotic cages instead since a smaller PI-LL mismatch need to be addressed. This can only be done by conducting the BEPO procedure on live patients.

An economical standing equilibrium in bipedal posture is influenced by many parameters, as described by Duval-Beaupere et al. In the clinical setting, PI is a more practical parameter than the IIA as PI can be measured on lateral radiographs [20]. Besides PI as a constant anatomical parameter, sacral slope (SS) and PT play an important role in analyzing spinal sagittal alignment (Fig. 3) [3]. These parameters are influenced by the position of the pelvis in space and could therefore not be assessed on the supine CT images in this study. Mathematically, PI, PT and SS can be linked by the formula: PI = PT + SS [2]. In patients with fixed flat back syndrome, a decrease of PI could reduce or completely nullify the PI-LL mismatch (PI-LL > 10) which might have a positive effect on the Health Related Quality of Life (HRQOL). Even more, due to the anatomical relation, we consider any decrease of PI in patients with fixed LL may lead to a decrease of PT, which improves the HRQOL as well [10, 21]. Surgical correction of PI-LL mismatch has also been associated with spontaneous improvement in knee flexion as compensation mechanism [22].

**Fig. 3:**
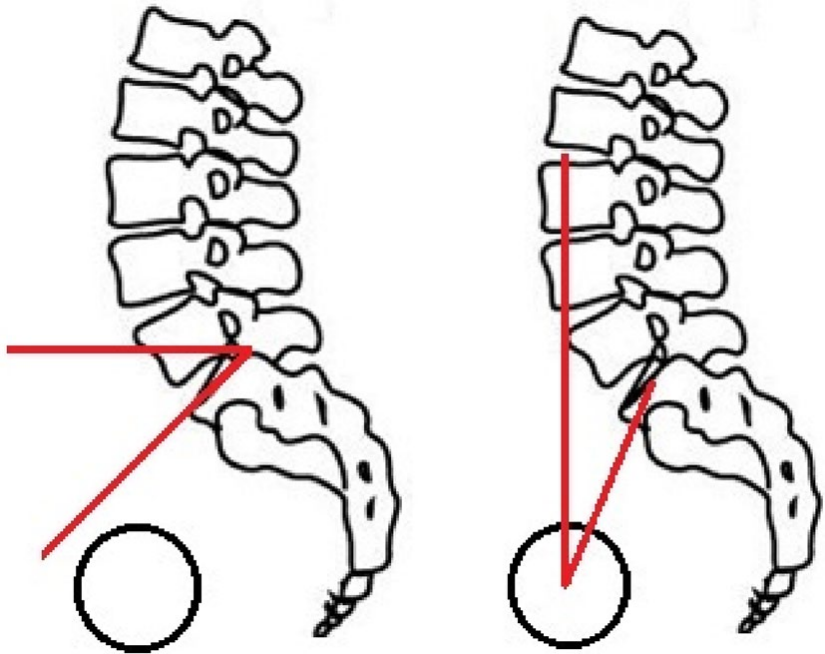
Left: sacral slope (SS): the angle between the superior plate of S1 and the horizontal line. Right: pelvic tilt (PT): the angle between the line connecting the center of the superior plate of S1 and the center of the femoral heads and the vertical line

In case a complex lumbar correction osteotomy such as a PSO is less appealing due to its known high risk of complications [23], previous spinal surgery, patients with severe sagittal malalignment could be eligible for this osteotomy. Clearly, the effects on restoration of spinal sagittal malalignment and HRQOL have to be confirmed by a prospective clinical series. However, simulation of the effect of BEPO on the sagittal vertical axis (SVA), defined as the distance between the C7 plumb line and the posterosuperior corner of S1 in the sagittal plane, in Surgimap^®^ (Nemaris Inc., New York, NY, USA) and found a promising decrease of 73.21 mm (48%) with an osteotomy angle of 10° (Fig. 4). Evenmore, this simulation was not able to correct for the flexion of the knees, so the actual effect on the correction of the sagittal malalignment and its concurrent compensatory mechanisms may even be more extensive.

**Fig. 4:**
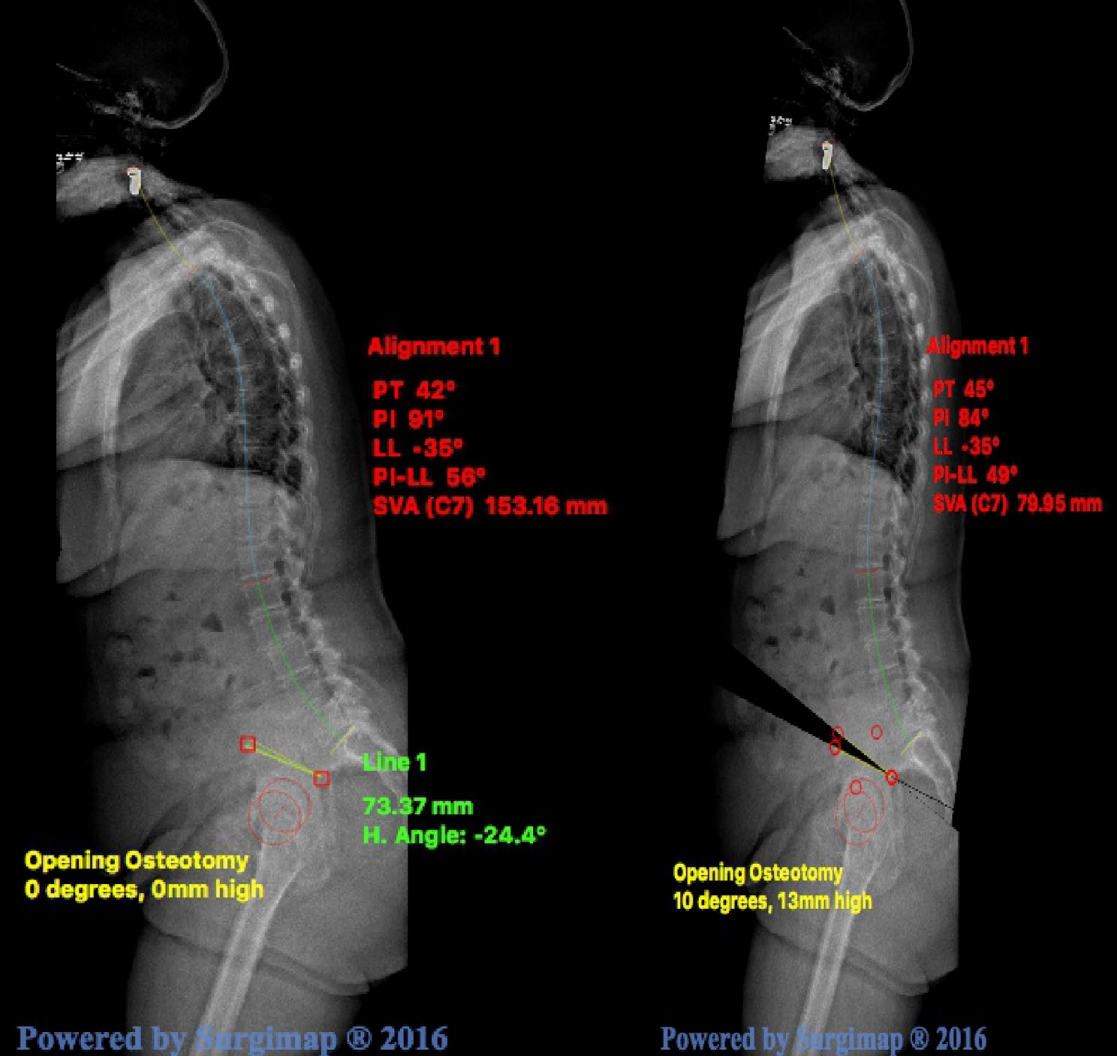
Simulation of effect on the sagittal vertical axis (SVA) after a bilateral extending pelvic osteotomy with an osteotomy angle of 15°. Pre-osteotomy (left): PI 91°, SVA 153.16 mm Post-osteotomy (right): PI 84°, SVA 79.95 mm

Technically, a bilateral osteotomy is not more demanding than a unilateral procedure. When the osteotomy is bilateral, the geometrical relation between sacrum and femoral heads is modified with a decrease of PI as consequence. CT simulation by Bodin et al. suggested that PI correction varies following a mathematical law: PI end = PI initial—*a* x (osteotomy angle). For the Salter osteotomy the value of *a* was 0.4964 and for the modified Salter osteotomy *a* was 0.3725, which was verified on a cadaveric model [16]. Using this formula to predict the PI decrease, using our osteotomy angle of 15°, we would, according to this formula, lead to a PI decrease of 0.4964 × 15 = 7.446° for the Salter and 0.3725 × 15 = 5.5875° for the modified Salter osteotomy. We found a substantially larger correction however, which might be explained by the specific modifications in the BEPO procedure. Our surgical technique is a modification of the original Salter osteotomy, with the most important modification being that the open-wedge was created with a saw cut through the arcuate line of the ilium, leaving a stable posterior cortex to act as a hinge. Also, Salter tested the pelvis by separating the halves in the mid-sagittal plane and fixing them on a rigid board to compare different osteotomies in the same specimen. We tested with the whole pelvis in the supine position. To our knowledge, Bodin et al. are the only other authors that have investigated the effect of an osteotomy of the ilium on PI in cadavers before. They focused on comparing different techniques rather than quantifying the impact of the osteotomy on PI. They also presented a small clinical series of patients who underwent bilateral (*n* = 8, of which 1 with additional psoas tenotomies) or unilateral (*n* = 3, of which one with contralateral closure and one with contralateral Chiari osteotomy) Salter innominate osteotomy. After a mean follow-up of 9.27 years (range 3–19 years), the mean PI reduction was 17°, which as an absolute number is far more than in our results. The mean preoperative PI of 74.6° in their study was relatively high. With a relative decrease of 23% compared to their PI, this is comparable to our data. SVA decreased from 97.2 mm to 50.2 mm (− 48.4%), which is almost within normal value (< 50 mm) and similar to our simulation. Patients had to use crutches for 6 weeks, wear a hemi-spica cast for 3 months and a lumbar orthosis for an additional 3 months. Nevertheless, many complications were reported, including persisting pain complaints (*n* = 10), hip disorders leading to total hip replacement (*n* = 3) and femoral neuropraxia (*n* = 1). In our cadaveric study, the osteotomy was complicated by a fracture with dislocation in specimen no. 4. We argue that the cut did not have the appropriate length, which increased the local stress on the posterior cortex during the bone distraction and resulted in the fracture which would have needed additional fixation for stability in the clinical setting. We then adjusted the surgical technique as described. In the sub analysis, we found a statistically significant decrease in both separate groups after adjusting the technique. The different groups did not differ in their effect on the correction of PI. Following this adjustment, no fissures nor fractures occurred with the same osteotomy angle. In a clinical setting, preoperative measurement of the total length of the ilium on CT scans would enable planning of the desirable saw length preoperatively and reduce the risk of such a complication. We decided not to exclude this specimen to prevent selection bias.

### Limitations

The most important limitation of this study is that it is a cadaveric study. Therefore, no conclusions could be made on global sagittal alignment nor the effect on soft tissues and muscles or concurrent postoperative pain. Also, we used a PLIF cage and no fixation plate in this study because the primary goal was to maintain the opening of the created wedge. In a clinical trial, a specifically designed cage that fits the patient-specific wedge perfectly with a fixation plate to provide pelvic stability should be used. A clinical trial will have to establish the effects and the clinical relevance. Because the study was set up as a proof of concept study, testing 10 cadavers was enough to answer the main question and find a statistically significant difference in PI.

### Future clinical implications

The clinical feasibility and relevance has yet to be established in a clinical trial. Although, the technical aspect of the surgery was found to be feasible in this cadaveric study. One of the main concerns for clinical implication is the risk of pseudoarthrosis. In a clinical trial, these patients should be preoperatively tested for osteoporosis. Also, a specifically designed cage should be used to improve bone ingrowth with a fixation plate to provide pelvic stability. This should be stable enough for patients to be able to mobilize with two crutches from the day after the surgery to 5–6 weeks after.

We speculate that this BEPO procedure may diminish the need for complex PSO surgeries with concurrent complications and increase the need for less complex procedures such as SPO or the use of hyperlordotic cages instead since a smaller PI-LL mismatch may need to be addressed. We do not believe BEPO will be a full alternative to complex spine surgeries but instead be considered as an extra tool in the armamentarium of the spine surgeon which may significantly reduce the extent of the spinal procedures in severely malaligned patients.

In conclusion, the correction of PI after a BEPO that was observed in this study was feasible and effective. We postulate that a BEPO could be an eligible tool to reduce the complexity of spinal procedures in patients with severe spinal sagittal malalignment. A prospective clinical series is required to further investigate the effects and safety in patients.

## References

[1] Boulay C, Tardieu C, Hecquet J (2006). Sagittal alignment of spine and pelvis regulated by pelvic incidence: standard values and prediction of lordosis. Eur Spine J.

[2] Legaye J, Duval-Beaupere G, Hecquet J (1998). Pelvic incidence: a fundamental pelvic parameter for three-dimensional regulation of spinal sagittal curves. Eur Spine J.

[3] Duval-Beaupere G, Schmidt C, Cosson P (1992). A barycentremetric study of the sagittal shape of spine and pelvis: the conditions required for an economic standing position. Ann Biomed Eng.

[4] Wang WJ, Crompton RH, Li Y (2003). Energy transformation during erect and 'bent-hip, bent-knee' walking by humans with implications for the evolution of bipedalism. J Hum Evol.

[5] Schlosser TP, Janssen MM, Vrtovec T (2014). Evolution of the ischio-iliac lordosis during natural growth and its relation with the pelvic incidence. Eur Spine J.

[6] Barrey C, Darnis A (2015). Current strategies for the restoration of adequate lordosis during lumbar fusion. World J Orthop.

[7] Schwab F, Patel A, Ungar B (2010). Adult spinal deformity-postoperative standing imbalance how much can you tolerate? An overview of key parameters in assessing alignment and planning corrective surgery. Spine (Phila Pa 1976).

[8] Aoki Y, Nakajima A, Takahashi H (2015). Influence of pelvic incidence-lumbar lordosis mismatch on surgical outcomes of short-segment transforaminal lumbar interbody fusion. BMC Musculoskelet Disord.

[9] Schwab FJ, Blondel B, Bess S (2013). Radiographical spinopelvic parameters and disability in the setting of adult spinal deformity: a prospective multicenter analysis. Spine (Phila Pa 1976).

[10] Ochtman AEA, Kruyt MC, Jacobs WCH (2020). Surgical restoration of sagittal alignment of the spine: correlation with improved patient-reported outcomes: a systematic review and meta-analysis. JBJS Rev.

[11] Kieser DC, Boissiere L, Cawley DT (2019). Validation of a simplified SRS-Schwab classification using a sagittal modifier. Spine Deform.

[12] Drazin D, Shirzadi A, Rosner J (2011). Complications and outcomes after spinal deformity surgery in the elderly: review of the existing literature and future directions. Neurosurg Focus.

[13] Salter RB (1966). Role of innominate osteotomy in the treatment of congenital dislocation and subluxation of the hip in the older child. J Bone Joint Surg Am.

[14] Colton CL (1972). Chiari osteotomy for acetabular dysplasia in young subjects. J Bone Joint Surg Br.

[15] Wilson PD, Levine DB (1969). Compensatory pelvic osteotomy for ankylosing spondylitis. A case report. J Bone Joint Surg Am.

[16] Bodin A, Roussouly P (2015). Sacral and pelvic osteotomies for correction of spinal deformities. Eur Spine J.

[17] Staubli AE, De Simoni C, Babst R (2003). TomoFix: a new LCP-concept for open wedge osteotomy of the medial proximal tibia–early results in 92 cases. Injury.

[18] Dubousset J (1990). CD instrumentation in pelvic tilt. Orthopade.

[19] Roussouly P, Gollogly S, Berthonnaud E (2005). Classification of the normal variation in the sagittal alignment of the human lumbar spine and pelvis in the standing position. Spine(Phila Pa 1976).

[20] Vrtovec T, Janssen MM, Pernus F (2012). Analysis of pelvic incidence from 3-dimensional images of a normal population. Spine (Phila Pa 1976).

[21] Lafage V, Schwab F, Patel A (2009). Pelvic tilt and truncal inclination: two key radiographic parameters in the setting of adults with spinal deformity. Spine (Phila Pa 1976).

[22] Cheng X, Zhang F, Wu J (2016). Spontaneous improvement of compensatory knee flexion after surgical correction of mismatch between pelvic incidence and lumbar lordosis. Spine (Phila Pa 1976).

[23] Daubs MD, Brodke DS, Annis P, Lawrence BD (2016). Perioperative complications of pedicle subtraction osteotomy. Global Spine J.

